# Effects of Long‐Chain n‐3 Fatty Acids Supplementation During Caloric Restriction on Body Composition in Overweight and Obese Adults: A Systematic Review and Meta‐Analysis of Randomized Controlled Trials

**DOI:** 10.1002/fsn3.70108

**Published:** 2025-04-08

**Authors:** Mansour Alblaji, Stuart R. Gray, Sophie Westrop, Dalia Malkova

**Affiliations:** ^1^ Human Nutrition, School of Medicine, Dentistry and Nursing, College of Medical, Veterinary, and Life Sciences University of Glasgow Glasgow UK; ^2^ Department of Basic Health Sciences, College of Applied Medical Sciences Qassim University Buraydah Saudi Arabia; ^3^ School of Cardiovascular and Metabolic Health, College of Medical, Veterinary and Life Sciences University of Glasgow Glasgow UK; ^4^ Institute of Sports Science and Innovation Lithuanian Sports University Kaunas Lithuania; ^5^ School of Health and Wellbeing, College of Medical, Veterinary and Life Sciences University of Glasgow Glasgow UK

**Keywords:** body composition, caloric restriction, long chain n‐3 PUFA, meta‐analysis, obesity, overweight

## Abstract

This systematic review aimed to determine whether caloric restriction‐induced reduction in body fat and fat‐free mass can be amended by supplementation with long‐chain n‐3 polyunsaturated fatty acids. Databases, including PubMed, Google Scholar, Web of Science, and EMBASE, were searched for papers published from the time the databases were created until November 1, 2023. Random‐effects model meta‐analyses were conducted using Review Manager 5.4.1 software. Statistical heterogeneity was assessed using the *I* ^2^. A standardized mean difference with a 95% confidence interval was calculated, and pooled effects were assessed. The initial search identified 1527 articles and 11 studies met the review inclusion criteria with 637 participants included. The participants' ages ranged between 18 and 61 years with a mean body mass index ranging between 27 and 36 kg/m^2^. The changes in fat‐free mass (standardized mean difference = 0.12, 95% CI −0.14 to 0.37, *p* = 0.36; *I* ^2^:35%) and fat mass (standardized mean difference = − 0.01; 95% CI −0.25 to 0.24; *p* = 0.96; *I* ^2^: 46%) were not different between intervention and control groups. The current review indicates that long‐chain n‐3 polyunsaturated fatty acids supplementation during caloric restriction neither attenuates the decline in fat‐free mass nor enhances the reduction in fat mass. Considering the small number of studies and interventions included, further research is needed to investigate the effectiveness of long‐chain n‐3 polyunsaturated fatty acids supplementation during caloric restriction.

## Graphical abstract

The present meta‐analysis indicates that LCn‐3 PUFA supplementation during caloric restriction does not enhance reductions in body mass and body fat, nor attenuate the decline in fat‐free mass.

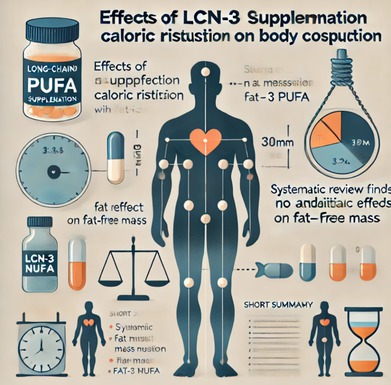

## 1 Introduction

Obesity, a global public health problem, is characterized by excessive fat accumulation and is associated with various adverse health outcomes, including type 2 diabetes, hypertension, respiratory disease, cardiovascular disease, and musculoskeletal issues (Kelly et al. 2008). The World Health Organization (WHO 2018) reported that the prevalence of obesity continues to rise (Golden 2024), particularly in developed countries (WHO 2018). This consistent trend is observed in adults across various socio‐demographic groups, lifestyle factors, and comorbidity statuses (Yang et al. 2023; Kim et al. 2024).

Caloric restriction (CR) is a key strategy to reduce body mass and confers protection against chronic diseases (Das et al. 2009; Harvie et al. 2011; Sundfor et al. 2018). However, CR‐induced body mass loss results in a decrease in both fat mass and fat‐free mass (FFM), with 25%–30% of body mass loss being related to the reduction in FFM (Turicchi et al. 2020; Chaston et al. 2007). This reduction in FFM is mainly related to a reduction in muscle mass (Armamento‐Villareal et al. 2014; Janssen and Ross 1999; Goodman et al. 1984; Weiss et al. 2017, 2007) and can, therefore, lead to reduced muscle strength and physical function (Santanasto et al. 2011). Furthermore, the extent of FFM reduction during CR‐induced weight loss is positively correlated with body mass regain during weight loss maintenance (Turicchi et al. 2020; Vink et al. 2016; Martins et al. 2022).

According to the previous evidence, supplementation with LCn‐3 PUFA, in the absence of CR or resistance exercise training, results in a significant increase in FFM (Noreen et al. 2010; Crestani et al. 2017). This beneficial impact of LCn‐3 PUFA on FFM can be attributed to several mechanisms. It has been reported that LCn‐3 PUFA stimulates muscle protein synthesis (Xu et al. 2022; Alkhedhairi et al. 2022; Dupont et al. 2019; Di Girolamo et al. 2014) enhances mitochondrial content and function, improves blood supply to skeletal muscle, and exerts anti‐inflammatory effects (Gray and Mittendorfer 2018). Supplementation with LCn‐3 PUFA, in the absence of CR, also reduces body fat (Noreen et al. 2010; Couet et al. 1997). Enhancing effects on serum adiponectin levels (Khorrami et al. 2020), whole‐body fat oxidation (Song et al. 2020), and energy expenditure at rest and during exercise (Logan and Spriet 2015; Yarizadeh et al. 2021) may be among the mechanisms responsible for the reduction in body fat due to LCn‐3 PUFA supplementation. Therefore, supplementation with LCn‐3 PUFA during CR may be a potential strategy to minimize the loss of FFM and thus muscle mass and function and facilitate body fat loss.

Two previous meta‐analyses, which included studies applying LCn‐3 PUFA supplementation combined with or without CR for 3 weeks or longer, reported that changes in FFM and body fat percentage were more favorable in the fish oil compared to the control groups (Bender et al. 2014; Alipour 2020). However, the impact of LCn‐3 PUFA supplementation during CR cannot be revealed from these two meta‐analyses (Bender et al. 2014; Alipour 2020). In addition, these meta‐analyses included studies conducted on lean and overweight/obese individuals living without and with existing diseases, including cardiovascular disease, cancer, and diabetes mellitus (Bender et al. 2014; Alipour 2020). Thus, our systematic review and meta‐analysis aimed to determine whether LCn‐3 PUFA supplementation during CR interventions lasting at least 8 weeks attenuates the CR‐induced decline in FFM and facilitates body fat loss in healthy individuals living with overweight and obesity.

## 2 Methods

The Preferred Reporting Items for Systematic Reviews and Meta‐Analyses (PRISMA) guidelines were used to report this systematic review (Page et al. 2021). This study was registered at the International Prospective Registry for Systematic Reviews (PROSPERO‐ CRD42021255309) (Data S1) (https://www.crd.york.ac.uk/prospero).

The search was conducted to identify relevant papers published from the time that the databases were created up to 1 November 2023. Two independent reviewers completed the literature search using the following databases: PubMed, Google Scholar, Web of Science, and EMBASE (Data S2). The following keywords were used “Omega 3,” “EPA,” “DHA,” “polyunsaturated fatty acids,” “PUFA” AND “Obesity,” “Obese,” “Adiposity,” “Adipose,” “weight,” “Overweight” AND “Calorie,” “Caloric restriction,” “Energy restriction,” “Weight loss diet,” “Dietary weight loss.” The terms “intervention,” “experiment,” “randomised clinical trial,” “controlled trial,” “blind,” and “placebo” were also used as search terms. In addition, hand searches of reference lists of identified key systematic reviews in the field and the reference lists of retrieved full‐text articles were also conducted. Citation screening through Google Scholar was performed for included full‐text articles as a final check. Only human clinical studies published in the English language were considered.

Articles were limited according to the following criteria, which were formulated based on the PICOS (population, intervention, comparison, outcomes, study design) method. Population (P): the participants must be adults living with overweight and obesity (BMI ≥ 25 kg/m^2^), in the absence of other major diseases such as cancer, diabetes, and heart disease, aged 18–65 years, men and/or women. Intervention (I): supplementation with LCn‐3 PUFA (EPA and DHA) during CR, and the supplements needed to be in the form of a capsule and consumed orally (not enteral or parenteral feeds) for at least 8 weeks or longer. The type of CR needed to be diet‐induced weight loss with a reduced energy intake. Comparison (C): CR alone or combined with a placebo supplement (control group). Outcomes (O): FFM, body fatness, body mass, and BMI. Study design (S): randomized controlled trials (RCTs).

The articles were electronically imported into Covidence (www.covidence.org), and duplicate studies were removed. The titles, abstracts, and full texts of the selected papers were screened by two authors. The screening was conducted according to the pre‐defined eligibility criteria. Points of disagreement were discussed, and a consensus was reached. A third reviewer arbitrated in case of disagreement. Reliability between reviewers for the title and abstract screening and full‐text screening was calculated in SPSS (version 28; SPSS IBM, New York, NY, USA) using Cohen's kappa scores, demonstrating almost perfect agreement and moderate agreement (*ĸ* = 0.88 and 0.67) for the title and abstract and full‐text article screening, respectively (Landis and Koch 1977).

The extraction of the data was obtained from included studies concerning participants, interventions, and outcomes. The data extraction fields included first author name, time and place of research, and general information; characteristics of study design (duration); characteristics of participants (gender, age, BMI categories); details of the intervention (quantity, dose, duration, and frequency); and outcomes (FFM, fat mass, body mass, and BMI). The outcomes were extracted as means and standard deviations (SDs) of change from baseline.

Risk of bias was conducted with the Revised Cochrane Risk of Bias tool for randomized trials (Risk of Bias 2) (Sterne et al. 2019). The Risk Of Bias 2 covered six domains: (1) selection bias (bias arising from the randomization process); (2) performance bias (bias due to deviations from intended interventions); (3) detection bias (bias due to missing outcome data); (4) attrition bias (bias in the measurement of the outcome); (5) reporting bias (bias in selecting the reported result); and (6) other bias. The risk of bias was considered low, high, or some concerns for each category. Two researchers independently conducted the risk of bias, and disagreement was resolved through discussion with a third reviewer.

Meta‐analyses were conducted using a random‐effects model in Review Manager (RevMan) version 5.4.1. The standardized mean difference (SMD) with a 95% confidence interval (CI) was computed as the effect size measures for each outcome between the treatment and control groups based on changes from baseline in mean and SDs and the sample size. For SMDs, the values were interpreted as low (≤ 0.2), moderate (0.3–0.5), and large (> 0.5) effect sizes (Schünemann 2021). Some studies reported only pre‐ and post‐intervention values, and, in these cases, the means and SDs for changes due to the intervention were calculated according to the formula provided in the Cochrane Handbook for Systematic Reviews of Intervention (Schünemann 2021).

Heterogeneity across studies was assessed using Cochrane's *Q* and *I* ^2^ statistics, with a significance level of *p* < 0.05, indicating evidence of statistical heterogeneity. The *I* ^2^ statistic describes the percentage of total variation across studies due to heterogeneity, with *I* ^2^ ≥ 50% indicating substantial heterogeneity (Higgins et al. 2003).

Publication bias was assessed through visual inspection of funnel plots of the effect size against the standard error of the effect size of the included studies and using Egger's linear regression approach (Egger et al. 1997). This method examines the association between effect size and standard error for each study and considers the sample size and effect size.

## 3 Results

**FIGURE 1:**
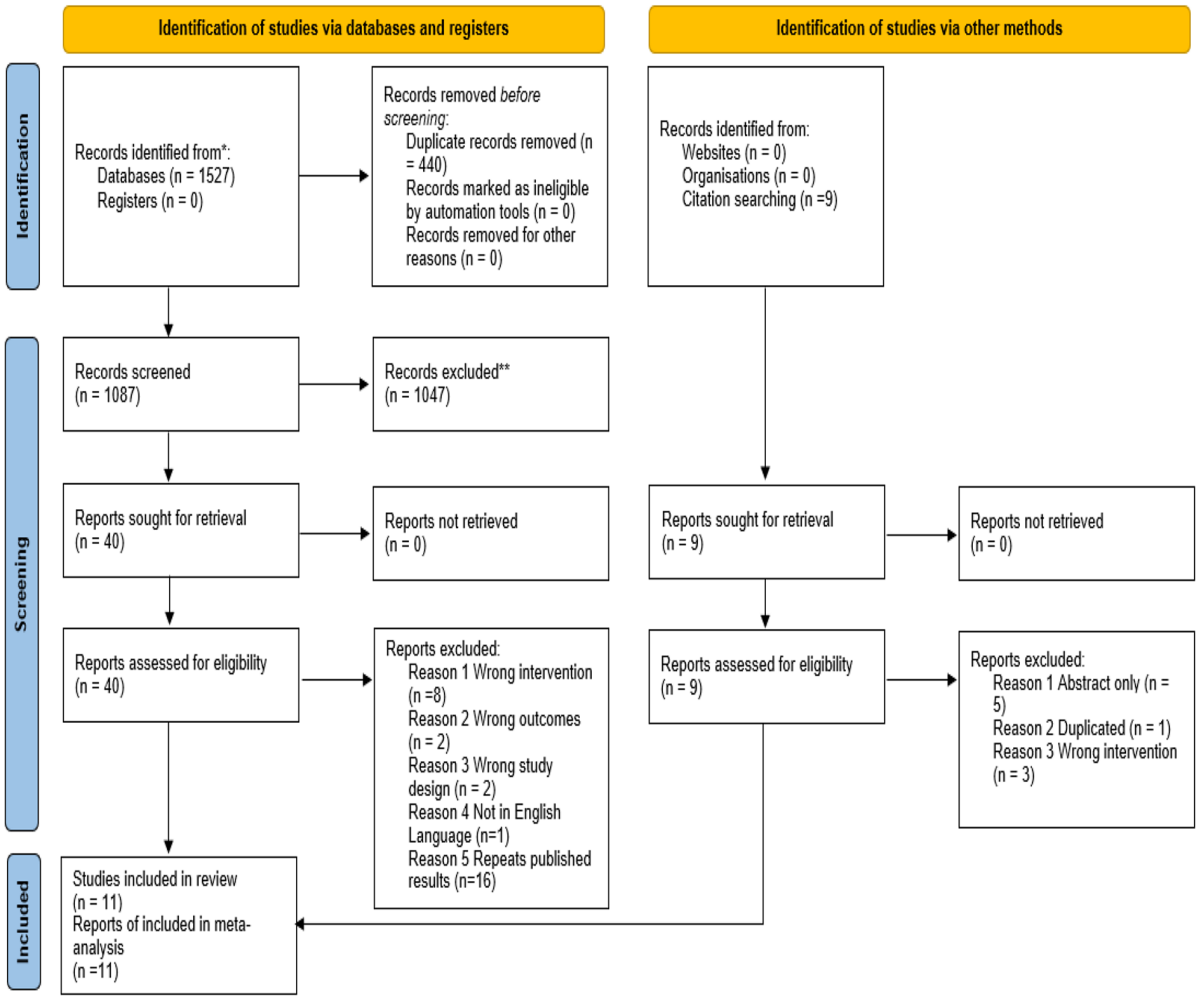
Flowchart of the studies selection process.

The searches of databases identified 1527 studies. Duplicate studies were removed, and initial title and abstract screening were conducted on 1078 studies. Nine further studies were identified from hand searching of reference lists. Following the screening of the titles and abstracts, full‐text screening was applied to 40 articles, and 11 articles met the eligibility criteria and were included in the final review (Figure 1). The complete list of excluded studies and the reason for exclusion are presented in the Data S3. The 11 eligible studies were extracted and assessed for the risk of bias assessment. These 11 studies were included in the meta‐analysis as they reported at least one of the research outcomes (FFM, fat mass, body mass, and BMI).

The details of the included studies are descriptively summarized in Table 1. Six studies were conducted in Europe (Krebs et al. 2006; Thorsdottir et al. 2007; Huerta et al. 2015; Razny et al. 2015; Romo‐Hualde et al. 2018; Salman et al. 2022), three in Australia (Munro and Garg 2013a; Wong et al. 2013, 2014), and two in Taiwan (Huang et al. 2018; Lee et al. 2015). The studies were published between 2006 and 2022. The number of participants across the included studies was 637, with sample sizes ranging from 12 to 160. Participants were healthy with an age range between 18 and 61 years. The lower mean BMI was 27 kg/m^2^ and the higher mean BMI was 36 kg/m^2^. The duration of the weight loss intervention with LCn‐3 PUFA supplementation ranged from 8 to 12 weeks. The dosage of LCn‐3 PUFA intake varied from 420 mg/day to 4000 mg/day, with EPA and DHA dosages ranging from 420 to 1300 mg/day and from 430 to 2900 mg/day, respectively. Only one study applied a higher dosage of DHA than EPA (Krebs et al. 2006).

**TABLE 1:** Characteristics of included studies.

Study (country)	Study population (*n*; gender, age, BMI)									
	Omega‐3	Control	Dietary intervention	Duration	Doses of LCn‐3PUFA	Body composition assessment	FFM	FM	BW	BMI
Huang et al. (2018) (Taiwan)	*N* = 48 overweight/obese (11 men and 37 women) Age: 20–40 years BMI: 29.4–35	*N* = 45 overweight/obese (14 men and 31 women) Age: 20–40 years BMI: 29.4–35	Intervention group: 2092–3347 KJ/day (CR) + 10 fish oil capsules/day Control group: 2092–3347 KJ/day (CR).	12 weeks	EPA: 1280 mg DHA: 850 mg	DEXA	**↓ FFM (#)	**↓ FM (#)	**↓ BW (#)	**↓ BMI (#)
Salman et al. (2022) (North Cyprus)	*N* = 20 overweight/obese Age: 30–60 years BMI: 27–35	*N* = 20 overweight/obese Age: 30–60 years BMI: 27–35	Intervention group: CR + 3 capsules/day (1020 mg n‐3 PUFA) Control group: CR	12 weeks	EPA: 580 mg DHA = 390 mg	Bioelectrical impedance	NR	**↓ FM (⇿)	**↓ BW (⇿)	**↓ BMI (⇿)
Huerta et al. (2015) (Spain)	*N* = 18 obese women Age: 20–50 years BMI: 27.5–40	*N* = 22 obese women Age: 20–50 years BMI: 27.5–40	Intervention group: 30% CR + 3 capsules/day (EPA: 1.3 g) Control group: 30% CR + 3 sunflower oil capsules	10 weeks	EPA: 1300 mg	DXA	FFM (⇿)	**↓ FM (⇿)	**↓ BW (⇿)	NR
Lee et al. (2015) (Taiwan)	*N* = 44 overweight/obese women Age: 37–63 years BMI: 24–35	*N* = 44 overweight/obese women Age: 37–63 years BMI: 24–35	Intervention group: 2092–3347 KJ/day (CR) + 10 fish oil capsules/day Control group: 2092–3347 KJ/day (CR).	12 weeks	EPA: 1280 mg DHA: 850 mg	DEXA	FFM (⇿)	FM (⇿)	BW (⇿)	BMI (⇿)
Thorsdottir et al. (2007) (Iceland, Ireland, (Spain)	*N* = 68 overweight (28 men and 40 women) Age: 20–40 years BMI: 27.5–32.5	*N* = 66 (24 men and 42 women) Age: 20–40 years BMI: 27.5–32.5	Intervention group: 30% CR + 6 fish oil capsules/day Control group: 30% CR + sunflower oil capsules	8 weeks	EPA: 633 mg DHA: 430 mg	Bioelectrical impedance	FFM (⇿)	**↓ FM (⇿)	**↓ BW (⇿)	**↓ BMI (⇿)
Razny et al. (2015) (Poland)	*N* = 24 obese (6 men and 18 women) Age: 25–65 years BMI: 30–40	*N* = 24 obese (4 men and 20 women) Age: 25–65 years BMI: 30–40	Intervention group: 5020–6276 KJ/day (CR) + 3 fish oil capsules/day Control group: 5020–6276 KJ/day (CR) + 3 corn oil capsules/day	12 weeks	1800 mg (DHA + EPA in a ratio of 5:1)	Bioelectrical impedance	NR	NR	**↓ BW (⇿)	**↓ BMI (⇿)
Romo‐Hualde et al. (2018) (Spain)	*N* = 15 obese women Age: 20–50 years BMI: 27.5–40	*N* = 19 obese women Age: 20–50 years BMI: 27.5–40	Intervention group: 30% CR + 3 capsules/day (EPA: 1.3 g) Control group: 30% CR + 3 sunflower oil capsules	10 weeks	EPA: 1300 mg	DXA	NR	**↓ FM (⇿)	NR	**↓ BMI (⇿)
Munro et al. (2013) (Australia)	*N* = 15 obese (5 men and 10 women) Age: 18–60 years BMI: 30–40	*N* = 18 obese (6 men and 12 women) Age: 18–60 years BMI: 30–40	Intervention group: 5000–6000 kJ/day (CR) + 6 fish oil capsules/day Control group: 5000–6000 KJ/day (CR) + 6 Sunola oil capsules/day	12 weeks	EPA: 420 mg DHA: 1620 mg	Bioelectrical impedance	FFM (⇿)	**↓ FM (⇿)	**↓ BW (⇿)	**↓ BMI (⇿)
Krebs et al. (2006) (UK)	*N* = 39 obese women Age: 21–69 years BMI: > 27	*N* = 38 obese women Age: 21–69 years BMI: > 27	Intervention group: 3347–3765 KJ/day (CR) + 5 fish oil capsules/day Control group: 3347–3765 KJ/day (CR) + 2.8 g linoleic acid and 1.4 g oleic acid	12 weeks	EPA: 1300 mg DHA: 2900 mg	DXA	NR	**↓ FM (⇿)	**↓ BW (⇿)	**↓ BMI (⇿)
Wong et al. (2013) (Australia)	*N* = 13 obese (6 men and 7 women) Age: 18–75 years BMI: > 30	*N* = 12 obese (7 men and 5 women) Age: 18–75 years BMI: > 30	Intervention group: 25% CR + 4 Omacor oil capsules/day Control group: 25% CR.	12 weeks	EPA: 46% DHA: 38%	Bioelectrical impedance	NR	**↓ FM (⇿)	**↓ BW (⇿)	**↓ BMI (⇿)
Wong et al. 2014 (Australia)	*N* = 13 obese (6 men and 7 women) Age: 18–75 years BMI: > 30	*N* = 12 obese (7 men and 5 women) Age: 18–75 years BMI: > 30	Intervention group: 25% CR + 4 Omacor oil capsules/day Control group: 25% CR.	12 weeks	EPA: 46% DHA: 38%	Bioelectrical impedance	↕ FFM (⇿)	**↓ FM (⇿)	NR	NR

Abbreviations: #, Indicates significant difference between groups; **↓, Indicates significant decrease in both groups; ⇿, Indicates no significant difference between groups; ↕, indicates no significant change from baseline in both groups; BMI, Body Mass Index (kg/m^2^); BW, body weight; CR, caloric restriction; DHA, docosahexaenoic acid; DXA or DEXA, dual‐energy x‐ray absorptiometry; EPA, eicosapentaenoic acid; FFM, fat‐free mass; FM, fat mass; FO, fish oil; KJ, kilojoules; NR, not reported; RCT, Randomized Controlled Trial.

The CR intervention varied across studies. Studies used either 25%–30% of the estimated total energy expenditure (Thorsdottir et al. 2007; Huerta et al. 2015; Romo‐Hualde et al. 2018; Wong et al. 2013, 2014) or applied a caloric deficit of 2092–6000 KJ/day (Krebs et al. 2006; Razny et al. 2015; Munro and Garg 2013a; Huang et al. 2018; Lee et al. 2015). Three studies applied an energy‐restricted diet of 30% less than the calculated total energy expenditure for a period of 8 and 10 weeks, respectively (Thorsdottir et al. 2007; Huerta et al. 2015; Romo‐Hualde et al. 2018). Two studies used a 25% energy‐restricted diet less than the participants' total energy expenditure for 12 weeks (Wong et al. 2013, 2014). Two studies applied 12 weeks of a deficit of 2092–3347 KJ/day, depending on the participants' regular daily dietary intake (Huang et al. 2018; Lee et al. 2015). The study by Razny et al. (2015) applied a 12‐week low‐calorie diet that contained an intake of 5020 and 6276 KJ/day for women and men, respectively (Razny et al. 2015). Another study used an energy‐reduced, portion‐controlled healthy eating weight loss diet (HEWLD) that contained an intake of 5000 KJ/day for females and 6000 KJ/day for males for 12 weeks (Munro and Garg 2013a). Twelve weeks of the energy‐restricted diet of 3347–3765 KJ/day were applied in another study (Krebs et al. 2006). One study reported that the diet was planned individually according to the subject's basal metabolic rate and physical activity, providing 55%–60% of daily energy from carbohydrates, 25%–30% from fats, and 12%–15% from proteins (Salman et al. 2022).

The type of LCn‐3 PUFA supplements varied among included studies. The majority of the studies used fish oil (Krebs et al. 2006; Thorsdottir et al. 2007; Razny et al. 2015; Munro and Garg 2013a; Huang et al. 2018; Lee et al. 2015), two studies used Omacor oil (Wong et al. 2013, 2014), and three studies did not report the source of LCn‐3 PUFA (Huerta et al. 2015; Romo‐Hualde et al. 2018; Salman et al. 2022). For placebo, three studies used sunflower oil (Thorsdottir et al. 2007; Huerta et al. 2015; Romo‐Hualde et al. 2018), one used Sunola oil (Munro and Garg 2013a), one study used linoleic acid (Krebs et al. 2006), one study used corn oil (Razny et al. 2015), and the rest of the studies used only CR without a placebo (Salman et al. 2022; Wong et al. 2013, 2014; Huang et al. 2018; Lee et al. 2015).

The adherence to the CR in the included studies was assessed in five studies (Thorsdottir et al. 2007; Salman et al. 2022; Munro and Garg 2013a; Wong et al. 2013, 2014). Two studies used a 24‐hour food recall (Wong et al. 2013, 2014), two studies used a 3‐day food record (Salman et al. 2022; Munro and Garg 2013a), and one study used 2‐day weighted food records (Thorsdottir et al. 2007). In some studies, the reduction in energy intake was 20% in CR with LCn‐3 PUFA and 24% in the control groups in three studies (Thorsdottir et al. 2007; Salman et al. 2022; Wong et al. 2013, 2014), but only slightly decreased in both groups (10.9% in CR with LCn‐3 PUFA and 6.7% in the control group) in one study (Munro and Garg 2013a). The other six studies did not report any data about adherence to the CR intervention (Krebs et al. 2006; Huerta et al. 2015; Razny et al. 2015; Romo‐Hualde et al. 2018; Huang et al. 2018; Lee et al. 2015).

Adherence to supplementation was assessed in seven studies (Thorsdottir et al. 2007; Razny et al. 2015; Munro and Garg 2013a; Wong et al. 2013, 2014; Huang et al. 2018; Lee et al. 2015). Two studies (Wong et al. 2013, 2014) used the return capsule count, and four studies used the proportion of the EPA, DHA, and LCn‐3 PUFA levels in the blood (Thorsdottir et al. 2007; Munro and Garg 2013a; Huang et al. 2018; Lee et al. 2015). One study used both methods (Razny et al. 2015). In two studies (Wong et al. 2013, 2014), a high adherence to LCn‐3 PUFA supplements was reported (> 95%), based on the capsule count. Five studies showed a significant increase in the proportion of EPA (0.68%) and DHA (0.68%) in the intervention group (fish oil) compared to the control groups, which indicated high compliance to the LCn‐3 PUFA supplements (Thorsdottir et al. 2007; Razny et al. 2015; Munro and Garg 2013a; Huang et al. 2018; Lee et al. 2015). The other four studies did not report adherence to the LCn‐3 PUFA supplements (Krebs et al. 2006; Huerta et al. 2015; Romo‐Hualde et al. 2018; Salman et al. 2022).

The rate of dropout in the included studies was reported in some studies. Nine studies reported the number of participants who dropped out of the study (Krebs et al. 2006; Thorsdottir et al. 2007; Huerta et al. 2015; Razny et al. 2015; Munro and Garg 2013a; Wong et al. 2013, 2014; Huang et al. 2018; Lee et al. 2015). In the study by Krebs et al. (2006), four participants (10.2%) dropped out in the intervention group and two in the control group (7.9%) (Krebs et al. 2006). In the study by Thorsdottir et al. (2007), 14 participants (17.5%) in the intervention group and 12 participants (15%) in the control group dropped out of the study (Thorsdottir et al. 2007). In the study by Huerta et al. (2015), nine participants (19%) dropped out in the control group, and two participants (10%) dropped out in the intervention group (Huerta et al. 2015). The number of participants who dropped out in a study by Munro and Garg 2013a) was seven (16%) in both groups (Munro and Garg 2013a). In the study by Wong et al. (2013), two participants (6%) dropped out of the study, without reporting from which group (Wong et al. 2013). In the study by Huang et al. (2018), two participants (4.2%) dropped out in the control group, and one participant (2%) dropped out in the intervention group (Huang et al. 2018). In the study by Razny et al. (2015), four participants (10%) dropped out in the intervention group and five participants (13.5%) dropped out in the control group (Razny et al. 2015). One study reported no dropout in the intervention group, while six participants (12%) dropped out in the control group (Lee et al. 2015). One study did not report the number of dropouts in participants; it only reported the number of participants who completed, but not the number who began the study (Romo‐Hualde et al. 2018). One study did not report the number of dropouts in participants; it only reported the number of participants who began the study (Salman et al. 2022).

**FIGURE 2:**
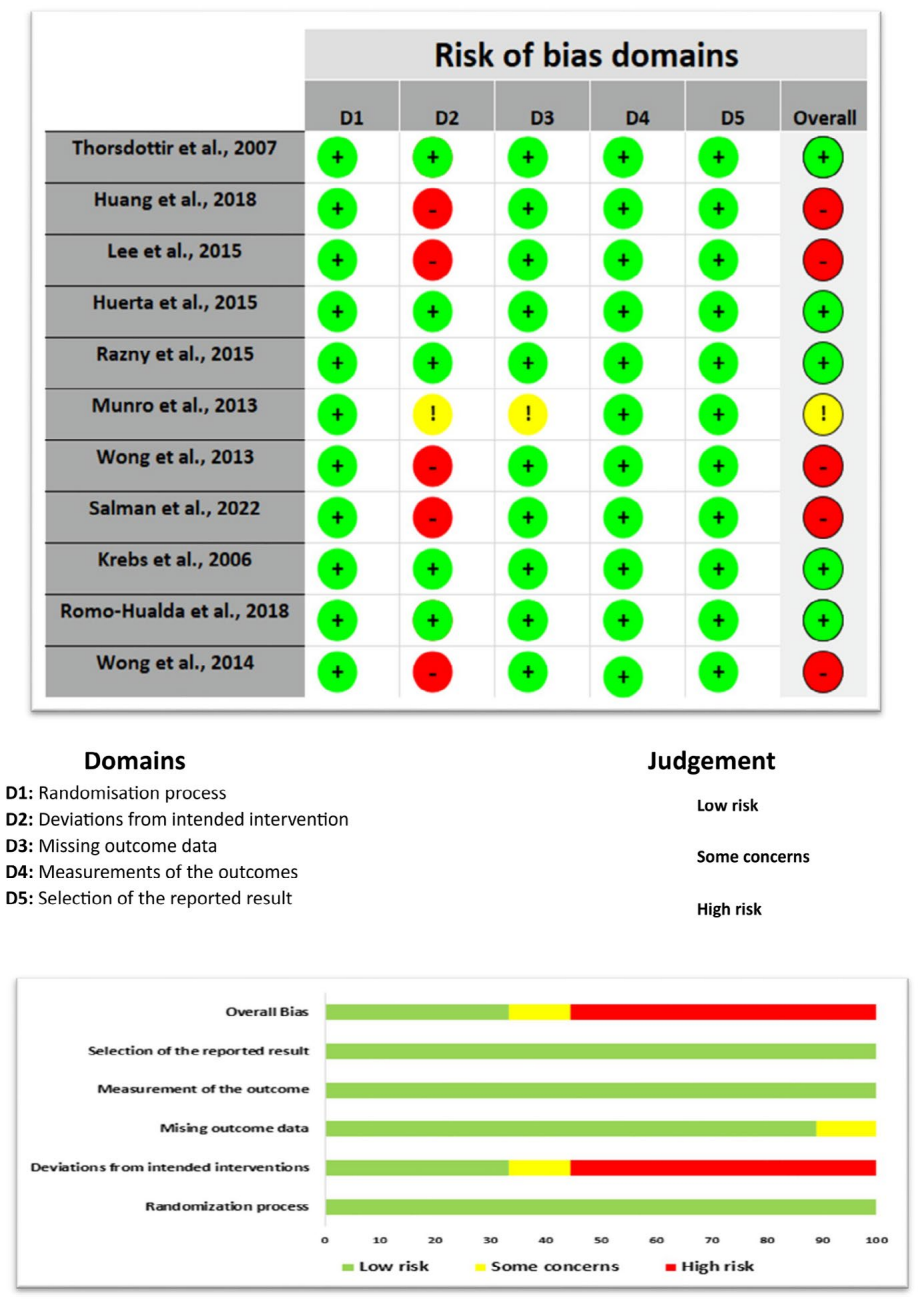
Cochrane risk of bias results for the included study.

The risk of bias for the included studies is presented in the Figure 2. Five of the 11 included studies (Salman et al. 2022; Wong et al. 2013, 2014; Huang et al. 2018; Lee et al. 2015) had a high risk of bias due to bias for deviations from the intended interventions, including not using a placebo in the control group (Salman et al. 2022; Wong et al. 2013, 2014; Huang et al. 2018; Lee et al. 2015), while one study (Munro and Garg 2013a) showed some concerns in the same domain. All trials were judged to be of low risk of bias for the randomization process (Domain 1), missing outcome data (Domain 3), measurement of the outcome (Domain 4), and selection of the reported result (Domain 5).

Regarding the power calculation of the included studies, two studies reported sample size calculations for one of the research outcomes (body mass loss) (Huerta et al. 2015; Huang et al. 2018). One study conducted sample size calculations for the primary outcome of insulin sensitivity (Razny et al. 2015). One study conducted sample size calculations for the primary outcome of changes in the Montreal Cognitive Assessment (MoCA) test scores (Salman et al. 2022). Seven studies did not report power or sample size calculations. Therefore, it is unclear if these studies achieved the power needed to detect a significant difference between groups in outcome measures.

Body mass and height in the included studies were measured to the nearest 0.1 kg and 0.1 cm, respectively. In the included studies (Krebs et al. 2006; Thorsdottir et al. 2007; Huerta et al. 2015; Razny et al. 2015; Salman et al. 2022; Munro and Garg 2013; Wong et al. 2013, 2014; Huang et al. 2018; Lee et al. 2015), height was measured with stadiometers, and the Tanita scale (Huerta et al. 2015; Razny et al. 2015; Salman et al. 2022; Wong et al. 2013, 2014; Huang et al. 2018; Lee et al. 2015) or a calibrated balance scale (Krebs et al. 2006; Thorsdottir et al. 2007; Munro and Garg 2013a) was used to measure body mass. Body mass and height were then used to calculate BMI (kg/m^2^).

The FFM and fat mass measurements were conducted using different techniques. In some studies, a bioelectrical impedance analysis (BIA) was used to measure FFM and body fatness (Thorsdottir et al. 2007; Razny et al. 2015; Salman et al. 2022; Munro and Garg 2013a; Wong et al. 2013, 2014), while the other five studies used DEXA scans (Krebs et al. 2006; Huerta et al. 2015; Romo‐Hualde et al. 2018; Huang et al. 2018; Lee et al. 2015).

**FIGURE 3:**
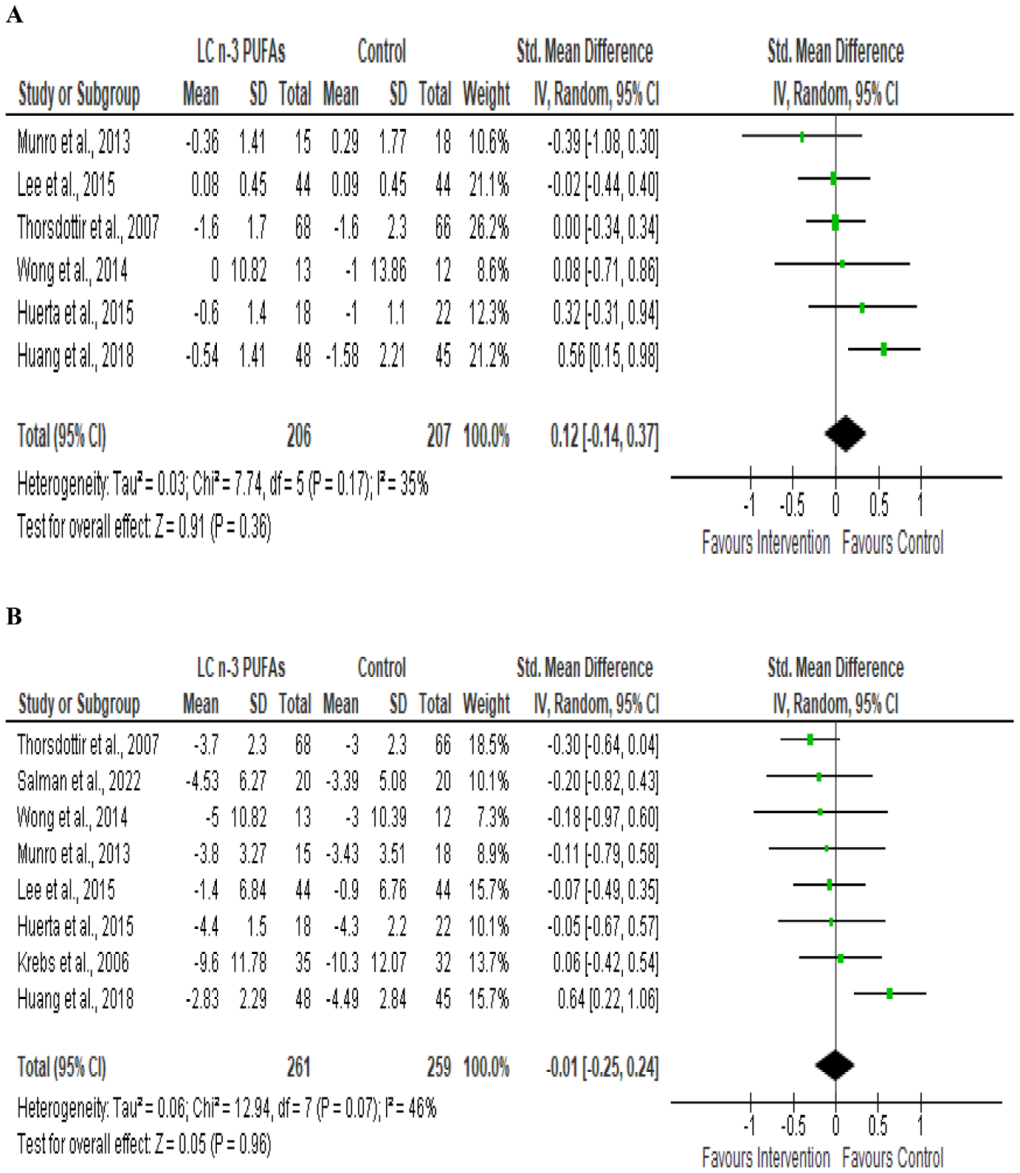
Forest plot of randomized controlled trials that compares the effects of the intervention (caloric restriction with LCn‐3 PUFA supplementation) and the control (caloric restriction alone or with Placebo) groups on fat‐free mass (A) and fat mass (B). Effects are presented as means and standard deviations (SD) for within‐group change from baseline and standardized (Std) mean differences with Random 95% CI. CI, confidence interval; df, degrees of freedom; IV, inverse variance; Z, weighted average effect size; Tau^2^, tau‐squared to estimate the variance between studies in a random‐effects meta‐analysis; Chi^2^, chi‐squared test (heterogeneity statistic) to test homogeneity; *I* ^2^, index of heterogeneity beyond within‐study sampling error.

A total of six studies, with 413 participants, measured the effect of supplementation with LCn‐3 PUFA during CR on FFM changes compared to the CR (Figure 3). The pooled effect size illustrated that FFM change between intervention and control groups was not different (SMD = 0.12; 95% CI −0.14 to 0.37; *p* = 0.36). There was no significant heterogeneity between studies (*I* ^2^ = 35%, *p* = 0.17).

Eight studies, with 520 participants, measured the effect of supplementation with LCn‐3 PUFA during CR on fat mass changes compared to the CR (Figure 3). The pooled effect size showed no effect on the change in fat mass (SMD = −0.01; 95% CI −0.25 to 0.24; *p* = 0.96). There was no significant heterogeneity between studies (*I* ^2^ = 46%, *p* = 0.07).

**FIGURE 4:**
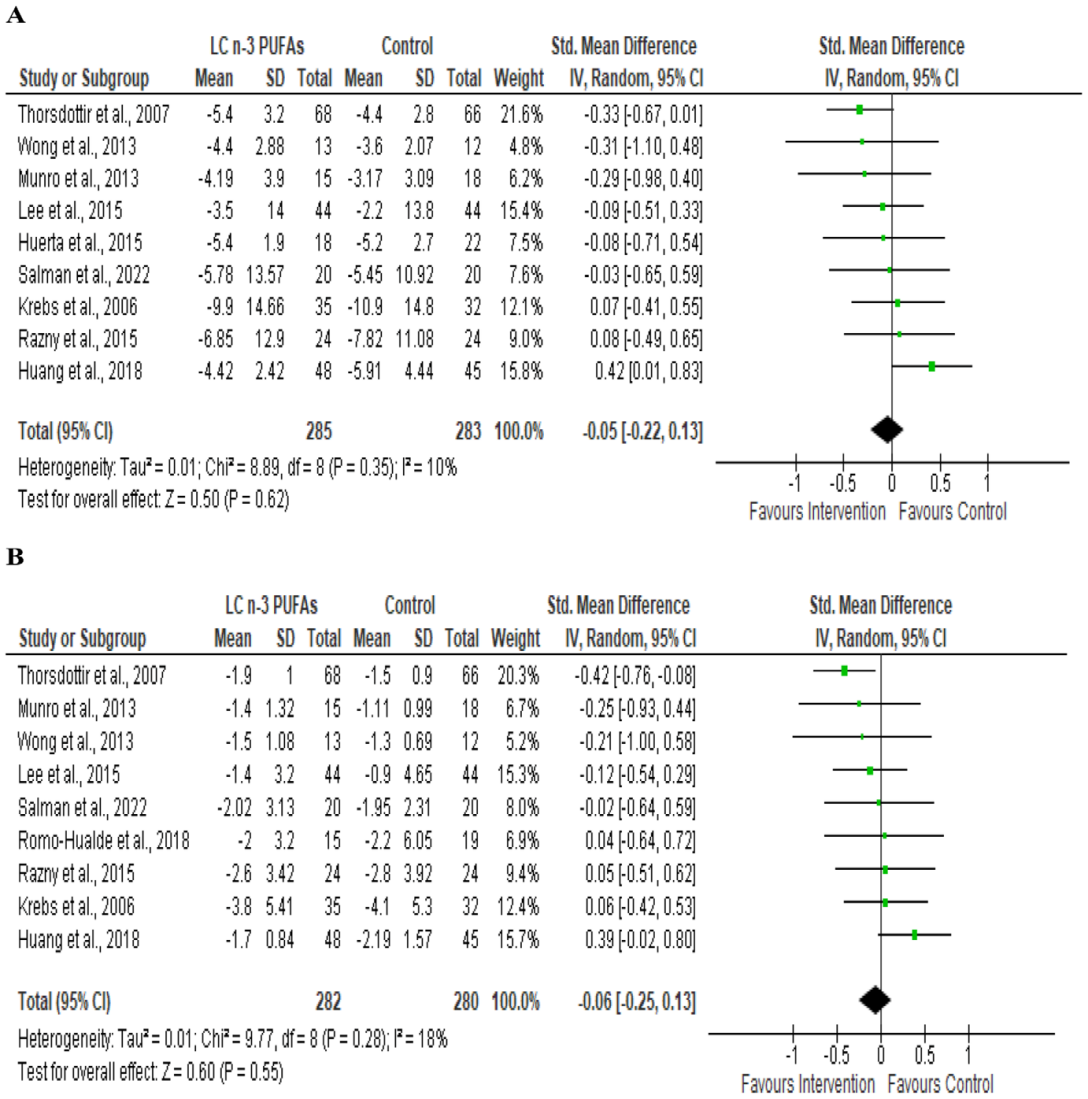
Forest plot of randomized controlled trials that compares the effects of the intervention (caloric restriction with LCn‐3 PUFA supplementation) and the control (caloric restriction alone or with Placebo) groups on body mass (A) and BMI (B). Effects are presented as means and standard deviations (SD) for within‐group change from baseline and standardized (Std) mean differences with Random 95% CI. CI, confidence interval; df, degrees of freedom; IV, inverse variance; Z, weighted average effect size; Tau^2^, tau‐squared to estimate the variance between studies in a random‐effects meta‐analysis; Chi^2^, chi‐squared test (heterogeneity statistic) to test homogeneity; *I* ^2^, index of heterogeneity beyond within‐study sampling error.

Nine studies, with 568 participants, measured the effect of supplementation with LCn‐3 PUFA during CR on body mass changes compared to the CR (Figure 4). The pooled effect size showed no effect on the change in body mass (SMD = −0.05: 95% CI −0.22 to 0.13; *p* = 0.62). No significant heterogeneity was reported between studies (*I* ^2^ = 10%, *p* = 0.35).

A total of nine studies, with 562 participants, measured the effect of supplementation with LCn‐3 PUFA during CR on BMI changes compared to the CR (Figure 4). The pooled effect size illustrated no effect on the change in BMI SMD = −0.06, 95% CI −0.25 to 0.13; *p* = 0.55. There was no significant heterogeneity between studies (*I* ^2^ = 18%, *p* = 0.28).

The funnel plots showed a potential risk of publication bias with FFM, fat mass, body mass, and BMI (Figures S1–S4).

## 4 Discussion

This is the first systematic review and meta‐analysis to investigate whether in adults living with overweight and obesity supplementation with LCn‐3 PUFA during CR lasting for at least 8 weeks benefits body composition changes. As expected, this systematic review of RCTs reported that CR‐induced body weight loss was related to body fat and FFM reductions. The novel finding of this systematic review was that supplementation with LCn‐3 PUFA during CR did not attenuate the loss of FFM nor enhance fat mass reduction. This finding, however, should be interpreted with caution due to the paucity of evidence and some studies having a high risk of bias.

Regardless of growing evidence that under conditions of sufficient energy provision, LCn‐3 PUFA has an anabolic effect on skeletal muscle metabolism (Robinson et al. 2018), including the rate of muscle protein synthesis (Dupont et al. 2019; Robinson et al. 2018; Smith et al. 2011), and increases FFM even without the application of resistance exercise (Noreen et al. 2010; Crestani et al. 2017; Logan and Spriet 2015), this systematic review found that in healthy individuals living with overweight and obesity, LCn‐3 PUFA supplements applied during CR lasting 8 weeks or longer did not attenuate the reduction in FFM. Therefore, the anabolic action of LCn‐3 PUFA under conditions of negative energy balance might be attenuated. On the other hand, a recent meta‐analysis reported that the application of LCn‐3 PUFA supplements under conditions of energy balance did not impact lean body mass (Cornish et al. 2022).

The lack of impact of LCn‐3 PUFA supplementation during CR on body fatness and body mass is somehow surprising since there is evidence suggesting that supplementation with LCn‐3 PUFA on its own increases energy expenditure at rest and during exercise and enhances fat oxidation rates (Logan and Spriet 2015; Yarizadeh et al. 2021) and acts as a ligand of energy metabolism‐related genes, which in turn leads to the up‐regulation of PPARγ and UCP2 expression (Moradi et al. 2021). Additionally, two previous meta‐analyses, which included studies with a minimum intervention duration of 4 weeks, reported that LCn‐3 PUFA supplementation facilitated body mass and body fat reduction (Bender et al. 2014; Alipour 2020). However, these systematic reviews did not distinguish if the effects were caused by LCn‐3 PUFA supplementation alone or LCn‐3 PUFA in conjunction with CR, with approximately only half of the studies included in these systematic reviews applying CR.

The lack of additional effects of LCn‐3 PUFA on body composition changes during CR interventions might be related to some of the limitations of the included studies. One limitation is that some of the included studies had a high risk of publication bias, which might have influenced the obtained results. Another limitation was the poor adherence to the CR among participants in one study (Munro and Garg 2013a) and the lack of investigation of adherence to CR in seven studies (Krebs et al. 2006; Huerta et al. 2015; Razny et al. 2015; Romo‐Hualde et al. 2018; Salman et al. 2022; Huang et al. 2018; Lee et al. 2015). Additionally, adherence to LCn‐3 PUFA supplements was assessed only in seven of the 11 studies. Also, differences in the level of CR, the amount of LCn‐3 PUFA prescribed, and the proportion of EPA and DHA in the provided supplements might have contributed to the different results obtained by individual studies.

We note that the findings of this systematic review are based on RCTs, most of which applied traditional fish oil capsules (Krebs et al. 2006; Thorsdottir et al. 2007; Razny et al. 2015; Munro and Garg 2013a; Huang et al. 2018; Lee et al. 2015), which are rich in EPA and DHA, and two studies used Omacor oil (Wong et al. 2013, 2014), a source of all three omega‐3 fatty acids (ALA, EPA, and DHA). It is important to note that the application of other sources of LCn‐3 PUFA, such as krill oil (Li et al. 2021), might be expected to benefit body composition changes during CR (Munro and Garg 2013a, 2013b, 2012; Thorsdottir et al. 2007; Lee et al. 2015; Huerta et al. 2015; Wong et al. 2014). Krill oil contains over 55% of its LCn‐3 PUFA in phospholipid form (Tou et al. 2007) and has some choline and astaxanthin, which are important in improving muscle quality and function (Moretti et al. 2020; Liu et al. 2018). Additionally, newly developed LCn‐3 PUFA supplements have been formulated, including triglyceride and ethyl ester forms with high concentrations of EPA and DHA (Fu et al. 2018). Furthermore, a novel high‐DHA tuna oil has already been investigated in animal studies, demonstrating its efficacy in attenuating obesity‐related features and metabolic dysfunctions in mice fed a high‐fat diet (Zhang et al. 2022). Therefore, the effects of these novel formulations on FFM preservation and body fat reduction warrant further investigation.

The finding of no beneficial effect on FFM changes may also be related to the lack of accuracy of body composition measurements. The measurement of FFM in the included studies was conducted using the bioelectrical impedance analysis technique (BIA) (Thorsdottir et al. 2007; Razny et al. 2015; Munro and Garg 2013a; Wong et al. 2013, 2014) or DEXA (Krebs et al. 2006; Huerta et al. 2015; Romo‐Hualde et al. 2018; Huang et al. 2018; Lee et al. 2015), which has been reported to provide low accuracy (overestimate FFM by an average of 2.36 kg and 2.09 kg in the case of BIA and DEXA, respectively) among obese individuals (Bosaeus et al. 2014). For instance, a study conducted by Jensen et al. (2019) showed that BIA overestimated FFM in obese individuals with a highly significant rate of systematic error (*p* = 0.001) (Jensen et al. 2019). In addition, FFM is only a proxy measure of muscle mass and thus lacks measurements of muscle mass; using techniques such as magnetic resonance imaging (MRI) (Jensen et al. 2019) means there is uncertainty in the effects of LCn‐3 PUFA supplementation on muscle mass during CR.

The limitation of our meta‐analysis is that only studies published in English were included; therefore, some relevant evidence could have been missed, which may cause a bias. This systematic review and meta‐analysis, however, have several strengths. Only randomized controlled trials were included, as they have a lower risk of bias than other types of studies (Lewis and Warlow 2004). It was also the first systematic review and meta‐analysis of RCTs that explored the possible effects of LCn‐3 PUFA supplementation during CR on FFM, fat mass, and body mass in overweight and obese individuals, with no other major health conditions.

Future research, employing LCn‐3 PUFA supplementation during CR, which includes larger sample sizes and a more robust methodology of body composition measurements, including direct measurements of muscle mass and muscle protein synthesis, would be needed. Consideration of muscle function measurement would also be important since there is evidence that supplementation with LCn‐3 PUFA can improve markers of muscle function, including lower body strength, time up‐and‐go, and 30s sit‐to‐stand performance independently of changes in body composition (Cornish et al. 2022). Supplementation with LCn‐3 PUFA has been shown to attenuate markers of metabolic syndrome and inflammation (Khan and Jackson 2018). Therefore, the inclusion of LCn‐3 PUFA supplementation in body weight management programs should be beneficial.

In conclusion, this systematic review and meta‐analysis of RCTs indicate that supplementation with LCn‐3 PUFA during calorie restriction lasting 8 weeks or longer does not attenuate the loss of fat‐free mass nor enhance fat mass reduction. Therefore, LCn‐3 PUFA supplementation appears to provide no significant benefits for body composition changes in the context of weight loss. Considering the small number of studies and interventions included, more research is needed to investigate the effects of LCn‐3 PUFA supplementation during CR on body composition modification, muscle strength, functional capacity, and muscle protein synthesis.

## Author Contributions

**Mansour Alblaji:** conceptualization (equal), data curation (lead), formal analysis (lead), investigation (lead), methodology (lead), project administration (equal), writing – original draft (equal), writing – review and editing (lead). **Stuart R. Gray:** conceptualization (equal), data curation (supporting), formal analysis (supporting), investigation (equal), methodology (equal), supervision (equal), writing – original draft (equal), writing – review and editing (equal). **Sophie Westrop:** formal analysis (supporting), methodology (supporting), writing – original draft (supporting), writing – review and editing (supporting). **Dalia Malkova:** conceptualization (lead), data curation (equal), formal analysis (supporting), investigation (lead), methodology (equal), software (equal), supervision (lead), validation (lead), visualization (supporting), writing – original draft (equal), writing – review and editing (lead).

## Conflicts of Interest

The authors declare no conflicts of interest.

## Supporting information


Data S1.

## Data Availability

The data that support the findings of this study are available from the corresponding author upon reasonable request.
