# Supplementary material for: Effects of Long‐Chain n‐3 Fatty Acids Supplementation During Caloric Restriction on Body Composition in Overweight and Obese Adults: A Systematic Review and Meta‐Analysis of Randomized Controlled Trials
Source: Food Sci Nutr. 2025 Apr 8;13(4):e70108. doi: 10.1002/fsn3.70108 (PMC11976066; doi:10.1002/fsn3.70108)
Supplement: Supplementary file 1 — Data S1. [file FSN3-13-e70108-s001.docx]

# Supplementary Materials

**Supporting Information 1:** Systematic review protocol- PROSPERO.

**Supporting information 2:** Searching strategy.

**Supporting information 3:** Reason for study exclusion. **Supporting Figure 1:** Funnel plot of changes in fat-free mass. **Supporting Figure 2:** Funnel plot of changes in fat mass.

**Supporting Figure 3:** Funnel plot of changes in body weight.

**Supporting Figure 4:** Funnel plot of changes in BMI.

**Supporting information 1:** Systematic review protocol- PROSPERO


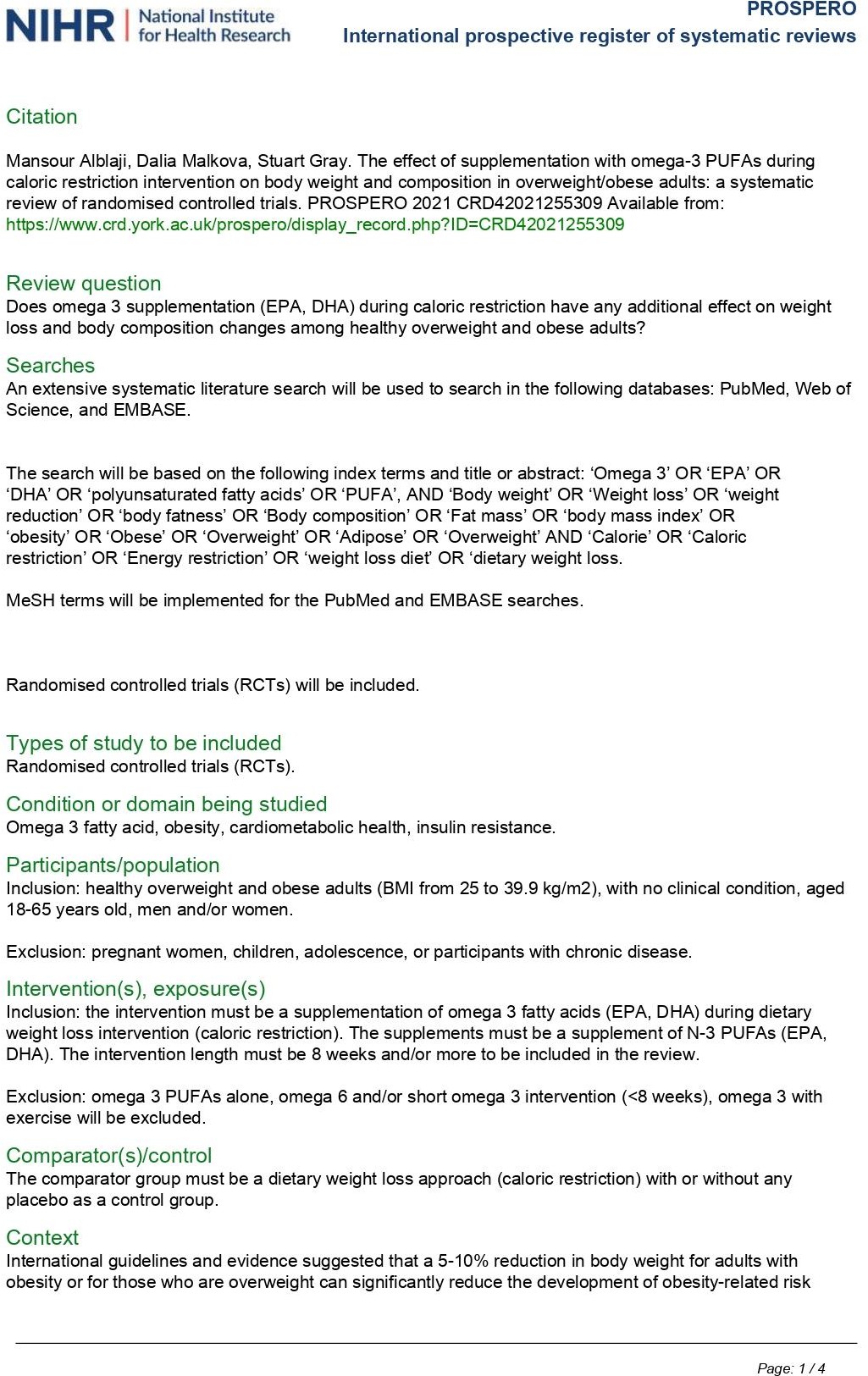


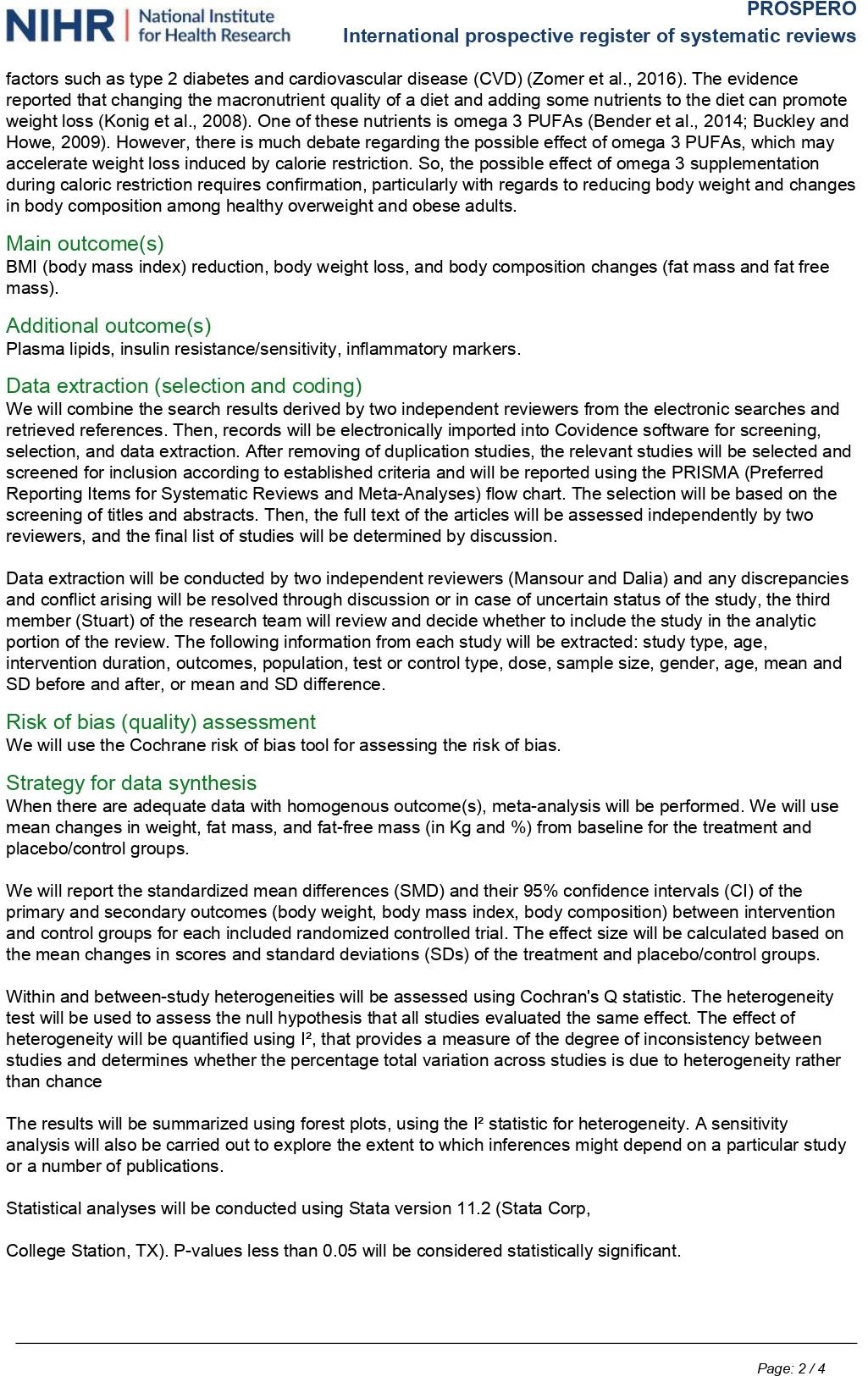


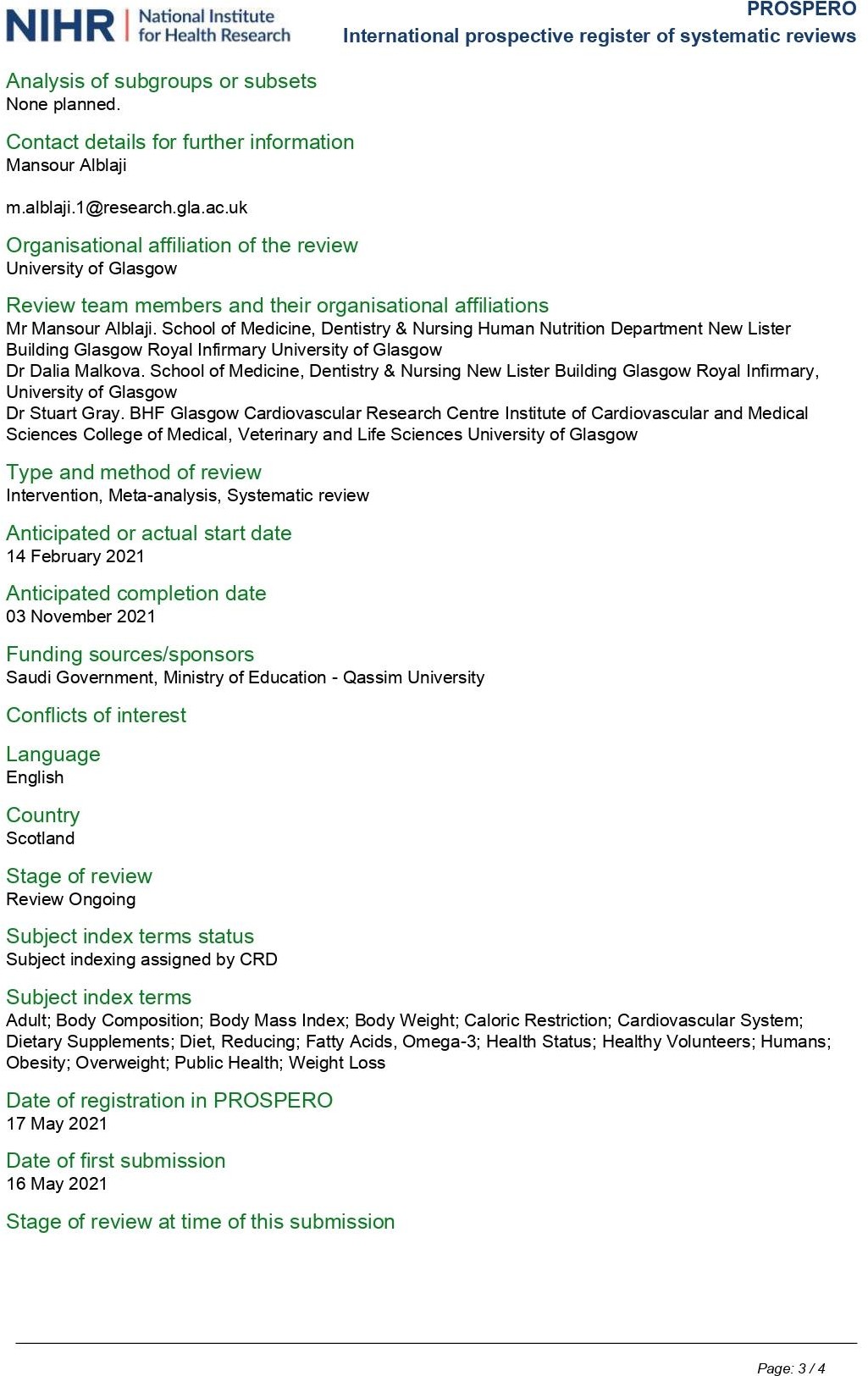


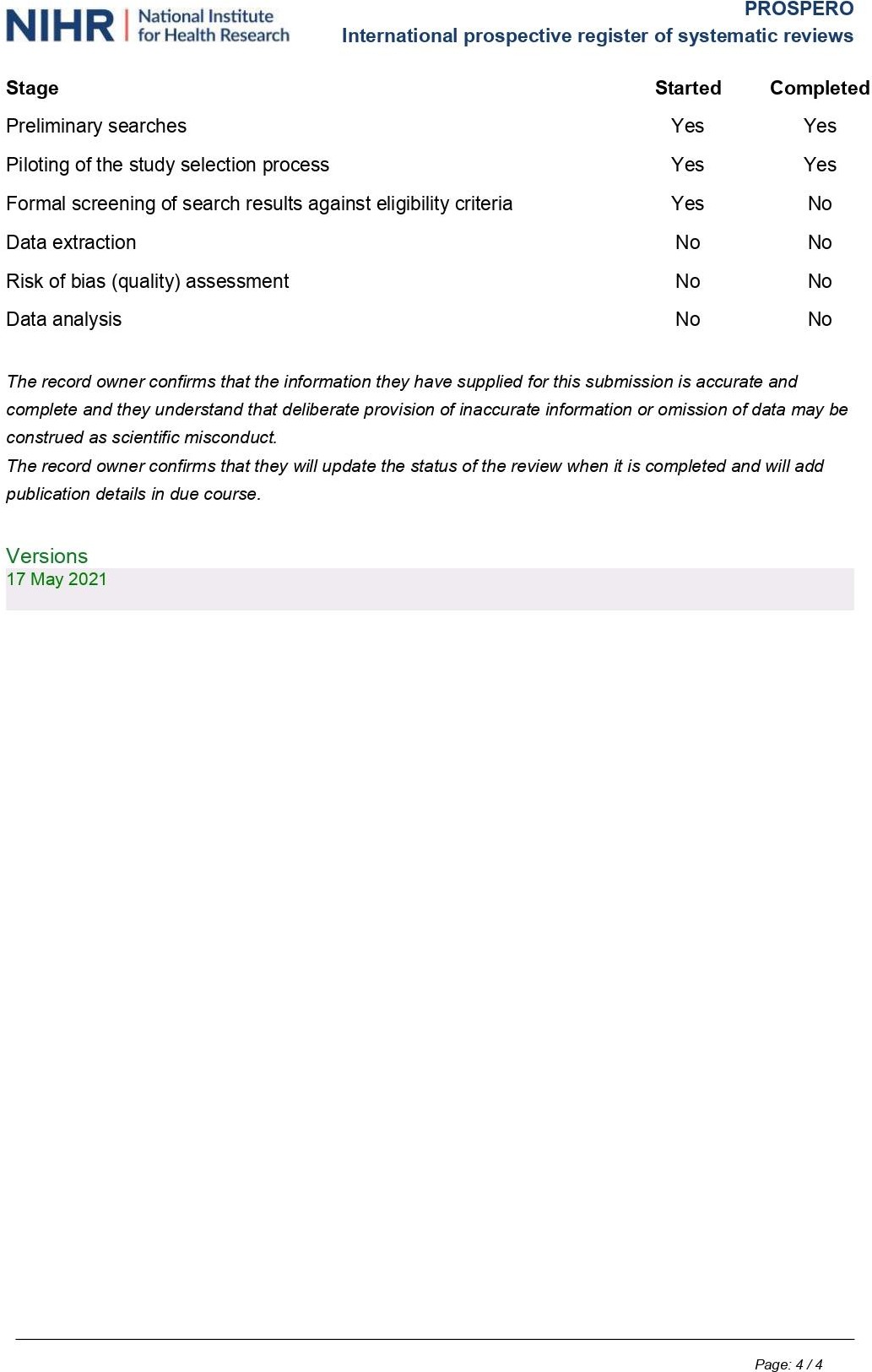


**Supporting information 2: Searching strategy**

An extensive systematic literature review was implemented, up to the 1^st^ of November 2023, using the following databases: PubMed, Google Scholar, Web of Science (ISI), and EMBASE.

**Searching strategy for each database**

| **Database** | **Keywords** |
| --- | --- |
| **PubMed** | ((("Omega 3"[Mesh Terms]) OR ("Fish oil" [Title/Abstract])) OR ("polyunsaturated fatty acids" [Title/Abstract])) OR ("EPA" [Title/Abstract])) OR ("DHA" [Title/Abstract])) OR ("PUFA" [Title/Abstract]))  AND ("weight loss" [MeSH Terms])) OR ("energy restriction " [Title/Abstract]). |
| **Google Scholar** | allintitle: Omega 3 polyunsaturated fatty acids OR obesity OR Obese OR adipose OR Adiposity OR weight  OR overweight OR calorie |
|  | allintitle: Omega 3 PUFA obesity OR Obese OR adipose OR Adiposity OR weight OR overweight OR calorie |
|  | allintitle: polyunsaturated fatty acids OR obesity OR Obese OR adipose OR Adiposity OR weight OR  overweight OR calorie ““““““Fish oil” “”””” |
| **Web of Science** | TITLE: (Omega 3) OR TITLE: (EPA, DHA) AND TITLE: (Weight) OR TITLE: (Overweight) OR TITLE:  (Obesity) AND TITLE: (energy restriction) Timespan: All years. Indexes: SCI-EXPANDED, SSCI, A&HCI, CPCI-S, CPCI-SSH, BKCI-S, BKCI-SSH, ESCI. |
| **EMBASE** | 'omega 3' exp/mj OR 'EPA' exp/mj OR 'DHA' exp/mj OR 'polyunsaturated fatty acids' exp/mj ) AND 'weight  loss'/exp/mj AND 'caloric restriction'/exp/mj) OR 'weight loss diet'/exp/mj |

The search was conducted based on the following terms and title or abstract:

1. Weight loss OR body weight OR body mass index OR body composition OR fat mass OR fat free mass OR boy fat OR overweight OR obese OR obesity OR adipose*.
2. Dietary weight loss approaches* OR weight loss intervention OR calorie restriction OR low energy diet OR very low energy diet OR low-calorie diet OR hypocaloric diet OR low fat/ high carbohydrate diet OR ketogenic diet.
3. Omega 3 fatty acid* OR fish oil* OR omega 3 polyunsaturated fatty acid OR ω-3 polyunsaturated fatty acid OR n-3 polyunsaturated fatty acid OR polyunsaturated fatty acid* OR eicosapentaenoic acid OR docosahexaenoic acid OR EPA OR DHA.
4. Clinical trial OR clinical trials OR clinic* trial* OR intervention OR RCT OR placebos OR placebo* OR random*.

**#1 AND #2 AND #3 AND #4**.

# Embase

1. exp omega 3/
2. (Omega-3 or fish oil or long chain omega 3 or EPA or DHA).tw.
3. (EPA or DHA or polyunsaturated fatty acids or pufa or pufas).tw.
4. exp caloric restriction/
5. (Caloric restriction* or weight loss diet* or energy restriction*).tw.
6. (Weight loss* Low-calorie diet* Very-low calorie-energy diet).tw.
7. Obesity.tw.
8. High BMI.tw.
9. Obese.tw.
10. Overweight.tw.
11. Adipose*.tw.
12. Omega 3 fatty acid*.tw.
13. fish oil*.tw.
14. n-3 PUFAs.tw.
15. ω-3 PUFA.tw.
16. EPA.tw.
17. DHA.tw.
18. Exp obesity/
19. Exp overweight/
20. eicosapentaenoic acid.tw.
21. docosahexaenoic acid.tw.
22. Weight reduction*.tw.
23. Body weight management.tw.
24. Weight management.tw.
25. Body weight.tw.
26. Body composition.tw.
27. Body fatness.tw.
28. Body fatness percentage.tw.
29. Fat mass.tw.
30. Fat-free mass.tw.
31. Body mass index.tw.
32. Weight loss approaches*.tw.
33. Energy restriction.tw.
34. Calorie restriction.tw.
35. Caloric restriction.tw.
36. Low-calorie diet.tw.
37. Low energy diet.tw.
38. Very-low calorie-energy diet.tw.
39. Hypocaloric diet.tw.
40. Randomised controlled trial.tw.
41. Controlled clinical trial.tw.
42. (randomized or randomised).tw.
43. randomi*.tw.
44. randomly.tw.
45. random allocation.tw.
46. trial.tw.
47. group.tw.
48. randomized trail.tw.
49. clinical-trail.tw.
50. control*.tw.
51. intervention stud*.tw.
52. (random* or alloc* or assign*).tw.
53. exp clinical trial/
54. (clin* adj3 trail*).tw.
55. 1 or 2 or 3 or 4 or 5 or 6 or 7 or 8 or 9 or 10 or 11 or 12 or 13 or 14 or 15 or 16 or 17 or 18 or 19 or 20 or 21

56. 22 or 23 or 24 or 25 or 26 or 27 or 28 or 29 or 30 or 31 or 32 or 33 or 34 or 35 or 36

1. 37 or 38 or 39 or 40 or 41 or 42 or 43 or 44 or 45 or 46 or 47 or 48 or 49 or 50 or 51 or 52 or 53 or
2. 55 and 56 and 57
3. Limit 58 to (human and English language)

# PubMed

1. exp Obesity/
2. exp High BMI /
3. exp Obese/
4. (Omega 3 fatty acid* or fish oil* or n-3 PUFAs * or ω-3 PUFAs* or EPA * or DHA or eicosatetraenoic acid or docosahexaenoic acid ).tw.
5. exp Weight loss approaches*/
6. exp Energy restriction/
7. exp Calorie restriction/
8. exp Low-calorie diet/
9. exp Very-low calorie-energy diet /
10. (Low caloric* adj3 (regime* or program*)).tw.
11. Weight reduction*.tw.
12. Body weight managementtw.
13. Body weight.tw.
14. Body fatness percentage.tw.
15. Randomized controlled trial.pt.
16. controlled clinical trial.pt.
17. (randomized or randomised).tw.
18. randomi*.tw.
19. randomly.tw.
20. Randomized-controlled trial.tw.
21. random allocation.tw.
22. trial.tw.
23. group.tw.
24. clinical-trial.pt.
25. clinical trial.tw.27. controlled-clinical-trial.pt.
26. cross-over-stud*.tw.
27. cross-over design*.tw.
28. cross-over-trial*.tw.
29. control*.tw.
30. intervention stud*.tw.
31. random-allocation*.tw.
32. (random* or alloc* or assign*).tw.
33. exp clinical trial/
34. (clin* adj3 trial*).tw.
35. 1 or 2 or 3 or 4 or 5 or 6 or 7 or 8 or 9 or 10 or 11 or 12 or 13 or 14
36. 15 or 16 or 17 or 18 or 19 or 20 or 21 or 22 or 23 or 24 or 25 or 26 or 27 or 28 or 29 or 30 or 31 or 32 or 33 or 34 or 35 or 36

37. 35 and 36

# Web of Science

1. TOPIC: ("Obesity")
2. TOPIC: ("High BMI")
3. TOPIC: ("obese”)
4. TOPIC: ("Overweight")
5. TOPIC: ("Adipose*")
6. TOPIC: ("Weight reduction*")
7. TOPIC: (Body weight management)
8. TOPIC: ("Weight management")
9. TOPIC: ((fit* low caloric/3 (regime* or program*)))
10. TOPIC: (“Body fatness percentage”)
11. TOPIC: (“Omega 3 fatty acid*”)
12. TOPIC: ("fish oil*)
13. TOPIC: ((“n-3 PUFAs”) or (“ω-3 PUFAs “))
14. TOPIC: ((“EPA”) or (“DHA “))
15. TOPIC: ("Weight loss approaches*")
16. TOPIC: ("Energy restriction")
17. TOPIC: ("Calorie restriction")
18. TOPIC: ("Low energy diet")
19. TOPIC: ("Very-low calorie-energy diet")
20. TOPIC: ("Hypocaloric diet ")
21. 1 or 2 or 3 or 4 or 5 or 6 or 7 or 8 or 9 or 10

22. 10 or 11 or 12 or 13 or 14 or 15 or 16 or 17 or 18 or 19

23. 21 AND 22

**Supporting information 3: Reason for study exclusion** (n=29)

# Reported same data and same experimental work (n=16)- these studies reported the same data, same experimental work and same results that had already been published in a primary study.

**These eight studies were reported in a primary study (Thorsdottir et al., 2007).**

- 1. Amaral CLD, Milagro FI, Curi R, Martínez JA. DNA Methylation Pattern in Overweight Women under an Energy-Restricted Diet Supplemented with Fish Oil. BioMed Research International. 2014;2014:1-10.
  2. Lucey AJ, Paschos GK, Cashman KD, Martínéz JA, Thorsdottir I, Kiely M. Influence of moderate energy restriction and seafood consumption on bone turnover in overweight young adults. The American Journal of Clinical Nutrition. 2008;87(4):1045-52.
  3. Ramel A, Jonsdottir MT, Thorsdottir I. Consumption of cod and weight loss in young overweight and obese adults on an energy reduced diet for 8-weeks. Nutrition, Metabolism and Cardiovascular Diseases. 2009;19(10):690-6.
  4. Ramel A, Martinez JA, Kiely M, Bandarra NM, Thorsdottir I. Moderate consumption of fatty fish reduces diastolic blood pressure in overweight and obese European young adults during energy restriction. Nutrition. 2010;26(2):168-74.
  5. Parra D, Bandarra NM, Kiely M, Thorsdottir I, Martínez JA. Impact of fish intake on oxidative stress when included into a moderate energy-restricted program to treat obesity. European Journal of Nutrition. 2007;46(8):460-7.
  6. Ramel A, Martinéz A, Kiely M, Morais G, Bandarra NM, Thorsdottir I. Beneficial effects of long- chain n-3 fatty acids included in an energy-restricted diet on insulin resistance in overweight and obese European young adults. Diabetologia. 2008;51(7):1261-8.
  7. Ramel A, Martinez JA, Kiely M, Bandarra NM, Thorsdottir I. Effects of weight loss and seafood consumption on inflammation parameters in young, overweight and obese European men and women during 8 weeks of energy restriction. Eur J Clin Nutr. 2010;64(9):987-93.
  8. Gunnarsdottir I, Tomasson H, Kiely M, Martinéz JA, Bandarra NM, Morais MG, et al. Inclusion of fish or fish oil in weight-loss diets for young adults: effects on blood lipids. International Journal of Obesity. 2008;32(7):1105-12.

# These four studies were reported in a primary study (Lee et al., 2015)

1. H-y-Su. A calorie-restriction diet supplemented with fish oil and high-protein powder is associated with reduced severity of metabolic syndrome in obese women. 2014.
2. Utami FA, Lee H-C, Su C-T, Huang S-Y. Calorie Restriction Supplemented with Fish Oil Ameliorates Abnormal Metabolic Status in Middle-Aged Obese Women: An Open-Label, Parallel-Arm, Controlled Trial. 2016.
3. Shabrina A, Tung T-H, Nguyen NTK, Lee H-C, Wu H-T, Wang W, et al. n-3 PUFA and caloric restriction diet alters lipidomic profiles in obese men with metabolic syndrome: a preliminary open study. European Journal of Nutrition. 2020;59(7):3103-12.
4. Utami FA, Lee H-C, Su C-T, Guo Y-R, Tung Y-T, Huang S-Y. Effects of calorie restriction plus fish oil supplementation on abnormal metabolic characteristics and the iron status of middle-aged obese women. Food & Function. 2018;9(2):1152-62.

# Two studies were reported in a primary study (Huerta et al., 2015)

- 1. Huerta AE, Prieto-Hontoria PL, Fernández-Galilea M, Escoté X, Martínez JA, Moreno-Aliaga MJ. Effects of dietary supplementation with EPA and/or α-lipoic acid on adipose tissue transcriptomic profile of healthy overweight/obese women following a hypocaloric diet. BioFactors. 2017;43(1):117-31.
  2. Huerta AE, Prieto-Hontoria PL, Sáinz N, Martínez JA, Moreno-Aliaga MJ. Supplementation with α-Lipoic Acid Alone or in Combination with Eicosapentaenoic Acid Modulates the Inflammatory Status of Healthy Overweight or Obese Women Consuming an Energy- Restricted Diet. J Nutr. 2015;146(4):889s-96s.

# One study was reported in a primary study (Krebs et al., 2006)

1. Mccombie G, Browning LM, Titman CM, Song M, Shockcor J, Jebb SA, et al. ω-3 oil intake during weight loss in obese women results in remodelling of plasma triglyceride and fatty acids. Metabolomics. 2009;5(3):363-74.

**One study was reported in a primary study (Razny et al., 2015)**

1. Razny, U., Goralska, J., Calder, P. C., Gruca, A., Childs, C. E., Kapusta, M., . . . Malczewska-Malec, M. (2022). The effect of caloric restriction with and without n-3 PUFA supplementation on bone turnover markers in blood of subjects with abdominal obesity: a randomized placebo-controlled trial. Nutrients, 13(9), 3096.

# Reason for exclusion: Wrong outcomes (n=2):

1. Parra, D., A. Ramel, N. Bandarra, M. Kiely, J. A. Martínez, and I. Thorsdottir. 2008. ‘““‘A diet rich in long chain omega-3 fatty acids modulates satiety in overweight and obese volunteers during weight ’loss’, *Appetite*, 51: 676-80.
2. Thorsdottir, I, Be Birgisdottir, M Kiely, Ja Martinez, and Nm Bandarra. 2009. ‘““‘Fish consumption among young overweight European adults and compliance to varying seafood content in four weight loss intervention ’diets’, *Public Health Nutrition*, 12: 592-98.

# Reason for exclusion: Wrong intervention (n=8):

1. Escoté, X., E. Félix-Soriano, L. Gayoso, A. E. Huerta, M. A. Alvarado, D. Ansorena, I. Astiasarán, J. A. Martínez, and M. J. Moreno-Aliaga. 2018. ‘““‘Effects of EPA and lipoic acid

supplementation on circulating FGF21 and the fatty acid profile in overweight/obese women following a hypocaloric ’diet’, *Food Funct*, 9: 3028-36.

1. Munro, Irene A., and Manohar L. Garg. 2012b. ‘““‘Dietary supplementation with n-3 PUFA does not promote weight loss when combined with a very-low-energy ’diet’, *British Journal of Nutrition*, 108: 1466-74.
2. Kriketos, A. D., R. M. Robertson, T. A. Sharp, H. Drougas, G. W. Reed, L. H. Storlien, and J. O. Hill. 2001. ‘““‘Role of weight loss and polyunsaturated fatty acids in improving metabolic fitness in moderately obese, moderately hypertensive ’subjects’, *J Hypertens*, 19: 1745-54.
3. Bao, Danny Q., Trevor A. Mori, Valerie Burke, Ian B. Puddey, and Lawrence J. Beilin. 1998. ‘““‘Effects of Dietary Fish and Weight Reduction on Ambulatory Blood Pressure in Overweight ’Hypertensives’, *hypertension*, 32: 710-17.
4. Tapsell, L. C., M. J. Batterham, K. E. Charlton, E. P. Neale, Y. C. Probst, J. E. ’’’’’O’Shea, R. L. Thorne, Q. Zhang, and J. C. Louie. 2013. ‘““‘Foods, nutrients or whole diets: effects of targeting fish and LCn3PUFA consumption in a 12mo weight loss ’trial’, *BMC Public Health*, 13: 1231.
5. Fontani, G., F. Corradeschi, A. Felici, F. Alfatti, R. Bugarini, A. I. Fiaschi, D. Cerretani, G. Montorfano, A. M. Rizzo, and B. Berra. 2005. ‘““‘Blood profiles, body fat and mood state in healthy subjects on different diets supplemented with Omega-3 polyunsaturated fatty ’acids’, *European Journal of Clinical Investigation*, 35: 499-507.
6. Plat, Jogchum, Annemarie Jellema, Julian Ramakers, and Ronald P. Mensink. 2007. ‘““‘Weight Loss, but Not Fish Oil Consumption, Improves Fasting and Postprandial Serum Lipids, Markers of Endothelial Function, and Inflammatory Signatures in Moderately Obese ’Men’, *The Journal of Nutrition*, 137: 2635-40.
7. Fisk, H.L., Childs, C.E., Miles, E.A., Ayres, R., Noakes, P.S., Paras-Chavez, C., Antoun, E., Lillycrop, K.A. and Calder, P.C., 2022. Dysregulation of subcutaneous white adipose tissue inflammatory environment modelling in non-insulin resistant obesity and responses to omega-3 fatty acids–a double blind, randomised clinical trial. Frontiers in Immunology, 13, p.922654.

# Reason for exclusion: Wrong study design (n=2):

1. Munro, Irene A., and Manohar L. Garg. 2013b. ‘““‘Prior supplementation with long chain omega-3 polyunsaturated fatty acids promotes weight loss in obese adults: a double-blinded randomised controlled ’trial’, *Food & Function*, 4: 650-58.
2. Paoli, Antonio, Tatiana Moro, Gerardo Bosco, Antonino Bianco, Keith Grimaldi, Enrico Camporesi, and Devanand Mangar. 2015. ‘““‘Effects of n-3 Polyunsaturated Fatty Acids (ω-3) Supplementation on Some Cardiovascular Risk Factors with a Ketogenic Mediterranean ’Diet’, *Marine Drugs*, 13: 996-1009.

**Reason for exclusion: No in English Language (n=1)- *reported in Spanish***

1. Marqués, M., D. Parra, M. Kiely, N. Bandarra, I. Thorsdottir, and J. A. Martínez. 2008. “““[Omega-3 fatty acids inclusion as part of an energy restricted diet to improve the effect on blood lipids]”””, *Med Clin (Barc)*, 130: 10-2.

-


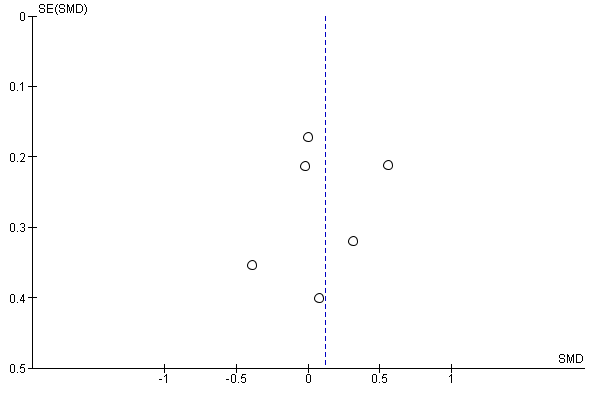


**Supporting Figure 1:** Funnel plot of changes in fat-free mass. SE= *standard error; SMD=* standardised mean difference.


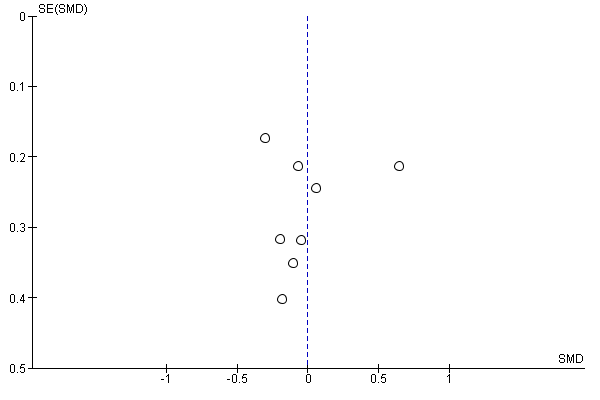


**Supporting Figure 2:** Funnel plot of changes in fat mass. SE= standard error; SMD= standardised mean difference.

**
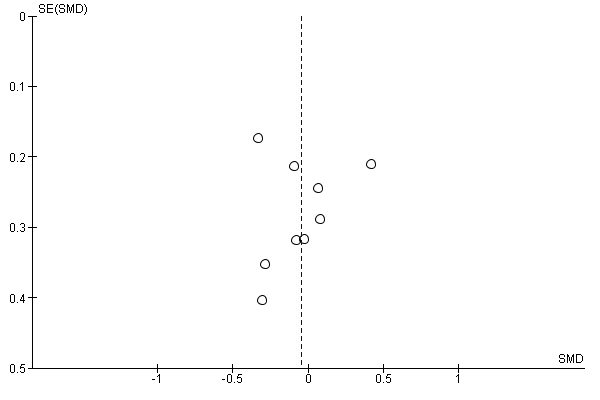
**

**Supporting Figure 3:** Funnel plot of changes in body weight. SE= standard error; SMD= standardised mean difference.


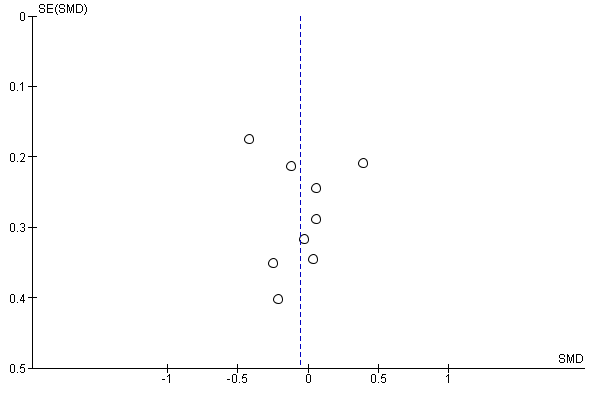


**Supporting Figure 4:** Funnel plot of changes in BMI. SE= standard error; SMD= standardised mean difference.
